# Multimodal Imaging and Macular Hyalocyte Count in a Patient with Acute Macular Neuroretinopathy

**DOI:** 10.1155/2022/2855191

**Published:** 2022-10-25

**Authors:** Michael J. Schatz, Oscar Otero-Marquez, Richard B. Rosen, Deep Parikh

**Affiliations:** The New York Eye and Ear Infirmary, 310 East 14th Street, New York 10003, USA

## Abstract

Though rare, acute macular neuroretinopathy is a well-described clinical entity. We report for the first time a detailed analysis of macular hyalocyte count and morphology during the acute phase of acute macular neuroretinopathy. We present a case of a 19-year-old man with bilateral acute onset paracentral scotomas in the setting of an antecedent viral infection. Multimodal imaging demonstrated classic features of acute macular neuroretinopathy. Further analysis revealed increased macular hyalocyte count and an activated hyalocyte morphology during the acute phase of the disease course. Multimodal imaging not only allows for greater confidence in a diagnosis and prognosis but also helps to shed light on underlying pathophysiology. The new clinical capability of monitoring cellular behavior, such as hyalocyte count and morphology, in physiological and pathological conditions may prove useful as biomarkers of early disease activity or response to therapy.

## 1. Introduction

Acute macular neuroretinopathy (AMN) is a rare condition characterized by acute onset paracentral scotomas with corresponding intraretinal reddish-brown, wedge-shaped lesions that tend to point toward the fovea [1, 2]. Although no causative etiology has discovered, known risk factors include antecedent upper respiratory or gastrointestinal infection, preeclampsia, oral contraceptives, and even excessive caffeine use [1]. Most characteristically, AMN demonstrates hyporeflective petalloid parafoveal lesions on infrared reflectance photography. Optical coherence tomography (OCT) is remarkable for distortion and hyperreflectivity of the outer retinal layers and disruption of the ellipsoid zone, with preservation of the retinal pigment epithelium (RPE). These features distinguish AMN from other conditions such as paracentral acute middle maculopathy (PAMM), which demonstrate inner layer hyperreflectivity, and solar retinopathy, which often demonstrates disruption of the photoreceptors as well as the RPE [3, 4]. Over time, these lesions tend to fade, and characteristic outer retinal layer hyperreflectivity gives way to outer retinal thinning [5]. More recently, these lesions have been shown to correspond to localized choriocapillaris flow voids seen on optical coherence tomography angiography [6]. Although many patients with AMN have improvement in their paracentral scotomas, complete resolution is atypical. No intervention has been shown to be definitively beneficial in this condition.

## 2. Case Report

A 19-year-old man presented to the emergency department with two days of progressive bilateral blurry vision. Two days prior to his symptoms, he had fever and cough and tested positive for influenza, negative for COVID-19, and had recently taken his second dose of oseltamivir (Tamiflu). On initial examination, he had count finger vision at one foot in both eyes, but with eccentric gaze, he was able to see 20/60 in each eye. Presence of improved visual acuity in eccentric gaze prompted Amsler grid testing which revealed bilateral inferior paracentral scotomas with no metamorphopsia. The patient was unable to read Ishihara color plates. Intraocular pressure, extraocular motility, and confrontation visual field testing were full. Pupils were equal, round, and reactive, with no relative afferent pupil defect. Anterior and posterior segment exams were remarkable only for asymmetric cup to disc ratio (0.6 OD and 0.3 OS). No macular or optic nerve head edema was seen. MRI brain and orbits with and without contrast were unremarkable.

**Figure 1:**
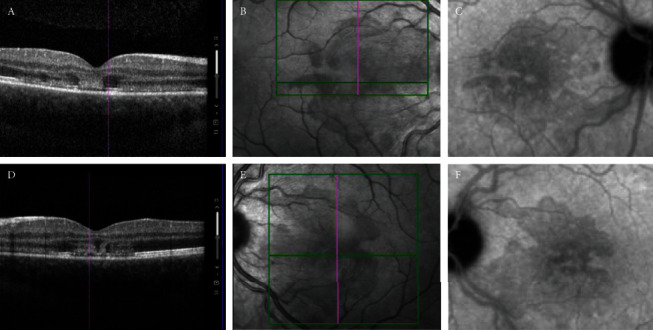
(a, d) Optical coherence tomography of the right and left eyes. Remarkable for distortion and hyperreflectivity of the outer retinal layers and disruption of the ellipsoid zone, with preservation of the retinal pigment epithelium (RPE). (b, e) Corresponding infrared reflectance photographs of the right and left eyes. Remarkable for hyporeflective macular lesions that correspond to areas of hypofluorescence seen in the indocyanine angiograms. (c, f) Late phase indocyanine green angiography of the right and left eye. Remarkable for mottled hypofluorescent macular lesions that correspond to the hypopreflective macular lesions seen on the infrared reflectance photographs.

On repeat examination two days later, best corrected visual acuity with noneccentric gaze had improved to 20/25 OD and 20/70 OS. The patient reported improvement in the paracentral scotomas. Dilated fundus exam was significant for mild macular edema and subtle red-brown foveal pigmentation notably not present at the time of initial. Optical coherence tomography showed outer retinal layer hyperreflectivity with distortion of the ellipsoid layer but intact retinal pigment epithelium (RPE) (Figures 1(a) and 1(d)). Hyporeflective macular lesions were seen most clearly on the infrared reflectance photographs (Figures 1(b) and 1(e)). Fluorescein angiography and fundus autofluorescence were within normal limits; however, indocyanine green angiography (Figures 1(c) and 1(f)) showed macular hypofluorescent lesions corresponding to the lesions seen on the infrared reflectance photographs.

**Figure 2:**
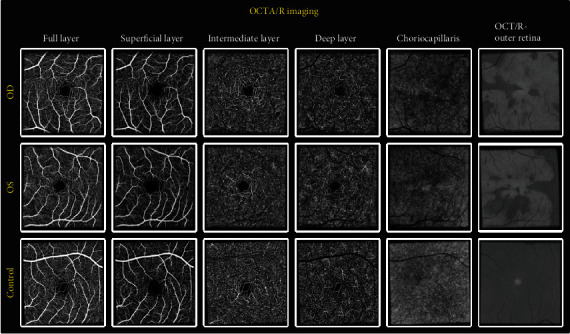
Optical coherence tomography angiography (OCT-A) of both eyes compared to healthy control, demonstrating choriocapillaris flow voids that roughly correspond to the overlying macular lesions seen on the infrared reflectance photographs, supporting the hypothesis that these represent areas of relative ischemia.

**Figure 3:**
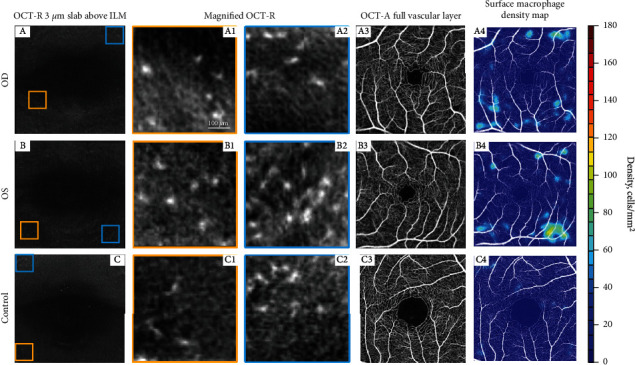
Surface hyalocyte distribution and morphology in right and left eye in our patient with AMN compared to 35-year-old healthy control, using en face OCT. (a–c) OCT-reflectance slab segmented 3 *μ*m above the ILM surface shows higher density of surface macrophage cells in eyes with AMN (more apparent on A4, B4, and C4). Orange and blue outlined images correspond to two different regions of interest with magnified view of surface macrophages (A1–C2). (A1–C2) Magnified view of surface macrophages in right and left eye in our patient with AMN and a healthy control. In the eyes with AMN, the cells appear round with fewer, shortened processes, consistent with in an “activated” state. In the healthy control, there are fewer cells and they appear slender with spindle- or star-like configuration consistent with a “quiescent” state. (A3, B3, C3) OCT-A full vascular layer of right and left eye in our patient with AMN and a healthy control. (A4, B4, C4) Overlay of hyalocyte density map onto the OCT-A. Surface hyalocyte density increases in acute ischemic conditions such as AMN. Cell density: AMN eyes, OD 9.26 cells/mm^2^, OS 10.38 cells/mm^2^, and control eye: 2.39 cells/mm^2^.

Optical coherence tomography angiography (OCT-A) imaging revealed choriocapillaris flow voids that roughly correspond to the lesions seen on infrared reflectance photographs (Figure 2). To assess retinal surface hyalocyte activity, a series of ten 3 × 3 mm scans centered at the fovea were acquired, registered, and averaged using a commercial spectral domain OCT system (Avanti RTVue-XR; Optovue, Fremont, CA, USA). Semiautomated macrophage identification and density measurements were performed on a 3 *μ*m OCT-Reflectance (OCT-R) image slab located above the inner limiting membrane (ILM) surface using MATLAB (2018b; MathWorks, Natick, MA). Relative to a 35-year-old healthy control, there was a notable increased macular hyalocyte count in our patient with AMN. The morphology of the surface hyalocytes was distinctively round with few processes, consistent with an “activated” phenotype (Figure 3).

## 3. Discussion

Hyalocytes are the primary resident macrophages within the vitreous. They act as the first responders to neuronal injury, playing an integral role in maintaining homeostasis in the retinal microenvironment, modulating the immune response, and synthesizing extracellular matrix [7–9]. They reside adjacent to the ciliary body and along the posterior pole localized according to histopathology to within the 50 *μ*m region above the inner limiting membrane (ILM), entangled with the collagen fibrils that compose the formed component of the vitreous cortex [10, 11].

Advances in retinal imaging have recently allowed visualization of vitreous cortex hyalocytes (VCH) in humans in vivo [12, 13]. Clinical OCT is capable of distinguishing phenotypic variation of VCH in response to changes in their activity. Under healthy conditions, hyalocytes appear primarily slender with a spindle-shaped or ramified configuration and distribute homogeneously. In retinopathic eyes, the cells transmorph into an “activated” phenotype which appears plumper with fewer, shorter processes, clustering around vessels and areas of metabolic disturbance in a nonuniform spatial distribution [13, 14].

During the acute inflammatory phase, our patient demonstrated classic features of AMN consistent with descriptions in the literature: outer retinal hyperreflectivity on OCT cross-sections, parafoveal petalloid hyporeflective lesions on infrared reflectance photography, corresponding areas of hypofluorescence on ICG, and the appearance of localized choriocapillaris flow voids in OCT-A scans. In this case, we were able to image the density, distribution, and morphology of VCH during the acute phase of AMN. Compared to the healthy control presented in this paper, as well as 18 other healthy controls previously published by Castanos et al. [13], this patient with AMN demonstrated increased VCH density, clustered around areas of metabolic disturbance as indicated by choriocapillaris flow voids, and the morphologic appearance of a more active phenotype. These findings are consistent with the hypothesis that the acute release of proinflammatory and proangiogenic cytokines and chemokines in ischemic conditions results in activation of VCH which attempt to restore the immunity and homeostasis of the retinal microenvironment. As the patient's symptoms began to show signs of resolution, the patient deferred follow-up, and we were unable to document the resolution of his hyalocyte count and morphology during recovery.

Refinement of imaging strategies makes it likely that similar events in the future will be able to be studied more extensively, enabling a more complete in vivo description of the cellular transformations as they develop. Such investigations will provide greater understanding of the cellular-level events occurring and possibly suggest therapeutic approaches.

## Data Availability

Data was taken and de-identified securely from a secure EMR within the Mount Sinai Health System.
